# Ibuprofen Rescues Abnormalities in Periodontal Tissues in Conditional *Presenilin 1* and *Presenilin 2* Double Knockout Mice

**DOI:** 10.3390/ijms140918457

**Published:** 2013-09-06

**Authors:** Jiansheng Su, Jiamei Gu, Zhuo Dong, Bing Mei

**Affiliations:** 1 Laboratory of Oral Biomedical Science and Translational Medicine, School of Stomatology, Tongji University, Middle Yanchang Road 399, Shanghai 200072, China; E-Mail: jiamei.gu@live.cn; 2 Shanghai Key Laboratory of Brain Functional Genomics, Key Laboratory of Brain Functional Genomics, Ministry of Education, East China Normal University, Shanghai 200062, China; E-Mail: dz820612@163.com

**Keywords:** ibuprofen, *presenilin*s, inflammatory response, periodontal tissues

## Abstract

We used forebrain-specific conditional *presenilin 1* (*PS1*) and *presenilin 2* (*PS2*) double knockout mice (dKO mice) that exhibit symptoms of neurodegenerative diseases, especially Alzheimer’s disease, to investigate whether ibuprofen can rescue brain and periodontal tissue abnormalities by attenuating the inflammatory response. Mandibles were dissected for alveolar bone-height analysis. Maxillae were fixed and decalcified for histological observation and osteoclast detection. ELISA measurements from the hippocampus, cortex, and gingiva of the mandibular incisor teeth were used to assay inflammatory mediators. We confirmed periodontal tissue abnormalities and inflammatory responses in brain and periodontal tissues in naive nine- and 12-month-old dKO mice. The other two groups of age-matched dKO mice that received 375-ppm ibuprofen treatment for six consecutive months exhibited significantly attenuated damage in periodontal tissues and reduction in several inflammation-related factors in brain and periodontal tissues. Our findings showed that the anti-inflammatory drug ibuprofen significantly decreased inflammation through the cyclooxygenase (COX) pathway in brain and periodontal tissues in dKO mice, and then attenuated abnormalities in periodontal tissues. This suggests that ibuprofen could be an ideal drug for preventing both nervous system and periodontal tissue damage caused by inflammatory responses.

## 1. Introduction

*Presenilin* mutations are linked to more than 90% of early onset familial Alzheimer’s disease (AD) cases [1]. *PS1*-knockout mice are embryonic lethal, while forebrain-specific *PS1*-knockout mice are almost normal, but lack enrichment-induced neurogenesis [2]. *PS2*-knockout mice however, are viable, fertile, and exhibit no obvious abnormal phenotypes [3]. Mice lacking both *PS1* and *PS2* in the forebrain (dKO mice) exhibit a series of age-dependent AD-like phenotypes, which include synaptic dysfunction, hyperphosphorylation of tau, and severe neurodegeneration [4–9], yet the level of Aβ (amyloid β) deposits are significantly reduced [8]. In terms of cognitive ability, mild spatial and contextual memory impairment emerges as early as two months and continues to progress, reaching quite serious levels when the dKO mice reach six months [5]. Besides these typical hallmarks, inflammatory changes also present in the dKO brain as an early and prominent feature [7,8]. Another group in our lab has confirmed that microglia and astrocytes are dramatically activated and complement C1qα and C4 are stimulated from an early age (three months) in the dKO brain. In addition, leukocyte serum levels were reported to be elevated beginning at six months, and cytokine and chemokine levels, such as those of TNF-α and IL-1β, were significantly higher both in brain and serum at nine months. These findings indicate that a robust inflammatory response appears early in the brains of dKO mice and then expands to the periphery [7].

Periodontitis is a lifelong, highly prevalent, peripherally infectious disease that is associated with gram-negative, anaerobic bacteria and is linked to localized and systemic inflammation. Gingival bleeding, loss of periodontal attachment and teeth are major markers of periodontitis [10]. Loss of periodontal attachment results in a decrease of alveolar bone height and exposure of the root surface [11]. Clinical studies have investigated the mutual influence between periodontitis and AD, and recent published studies show that elder adults with periodontitis or with several missing teeth exhibit more cognitive impairment compared with those without periodontitis or with few missing teeth [12–15]. According to other studies, poor cognition or cognitive decline may also lead to oral health issues such as tooth loss, caries, and poor plaque control [16–18]. Based on studies of dKO mice, it has been found that inflammatory responses in dKO mice brains can expand to oral tissue. The number of osteoclasts and amount of alveolar bone loss significantly increased at nine months, periodontal tissues showed obvious histomorphological abnormalities, and moreover, inflammatory mediators—such as TNF-α and IL-1β*—*increased in gingiva with age. These data indicate that inflammation in dKO mice brains can expand to the periphery and finally lead to abnormalities in periodontal tissues [19].

Novel clinical and biological evidence has engendered renewed interest regarding strategies targeting the inflammatory processes and, in particular, on the potential use of non-steroidal anti-inflammatory drugs (NSAIDs) in preventing or treating AD. There is compelling epidemiological evidence that long-term NSAID therapy delays the onset of AD [20] and reduces one’s lifetime risk of developing AD [21,22]. In addition, numerous experimental and clinical studies suggest NSAIDs as a therapeutic possibility for preventing alveolar bone resorption as periodontitis progresses [23]. Ibuprofen was one of the most frequently used anti-inflammatory drugs in those studies, and it may reduce the severity of inflammatory responses both in periodontitis and AD because it can cross the blood-brain barrier [21,23]. Here, we therefore examined whether six months of ibuprofen treatment could suppress inflammatory responses in the brain at three months, when inflammatory responses first appear in the *PS*-dKO mice, and at six months, when inflammatory responses expand to the periodontal tissue. We administered ibuprofen for six months because research indicates that reductions in IL-1β and insoluble Aβ depend on the treatment duration (6 months > 3 months) [24,25]. Moreover, this allows us to discuss potential therapeutic methods that might attenuate abnormalities in the brain and periodontal tissues.

## 2. Results and Discussion

### 2.1. Ibuprofen Decreased Alveolar Bone Loss of the Mandibular Molar Region in *PS*-dKO Mice

**Figure 1:**
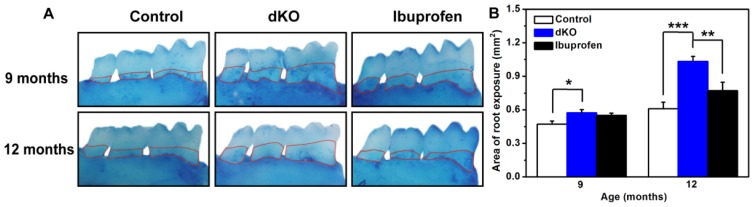
Alveolar bone loss (**A**) Mandibular lingual aspects of control mice at nine and 12 months of age (9 months *n* = 7, 12 months *n* = 9), naive double knockout mice (dKO mice) (*n* = 9), and ibuprofen-treated dKO mice (9 months *n* = 8, 12 months *n* = 9). The area enclosed by the red line is the exposed molar root area; (**B**) Root exposure area (mm^2^) of control, naive dKO, and ibuprofen-treated dKO mice at nine and 12 months of age. Values are mean ± SE for each group. *p < 0.05, **p < 0.01, ***p < 0.001.

The periodontal attachment loss is a major marker of periodontal destruction, but it is difficult to quantify the width of the periodontal membrane. However, decrease in alveolar bone height and exposure of the root surface as a result of periodontal attachment loss are easily quantified [10]. The development of alveolar bone loss in naive dKO mice was much faster than in control mice (Figure 1A). Further, although the amount of root exposure in naive and ibuprofen-treated dKO mice did not significantly differ at nine months, at 12 months it was significantly reduced (*p* < 0.05) in naive dKO mice (Figure 1B).

### 2.2. The Number of Osteoclasts in the Periodontal Ligament Was Significantly Less in Ibuprofen-Treated Mice

**Figure 2:**
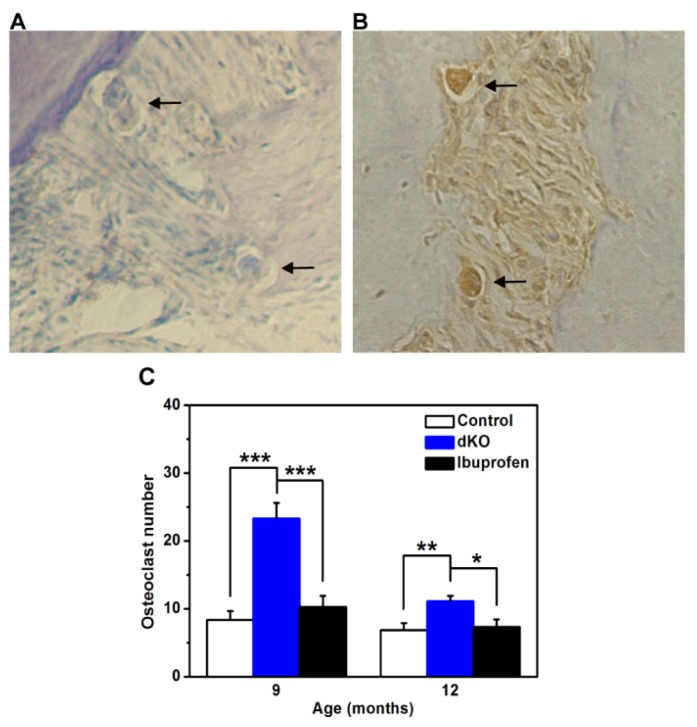
Representative examples of immunostaining of osteoclasts in periodontal ligaments of naive dKO mice. (**A**) Negative control for CTR immunostaining in periodontal ligament; (**B**) CTR-positive osteoclasts and resorption lacunae in periodontal tissue. Magnification for A and B is 400×; (**C**) The number of osteoclasts. CTR-positive cells in the periodontal ligament from five sections of each animal were counted. Values are mean ± SE. There were a significantly greater number of osteoclasts (CTR-positive cells) in naive dKO mice (*n* = 9) compared with control mice (*n* = 7–9) and ibuprofen-treated dKO mice (*n* = 8–9). *p < 0.05, **p < 0.01, ***p < 0.001.

The loss of connective tissue and bone in periodontal tissue is largely caused by the activation of osteoclasts and matrix metalloproteinases [10]. Osteoclasts differentiate from macrophage precursor cells in periodontal ligaments to absorb bone [26]. Therefore, we counted the number of osteoclasts (CTR-positive cells) in the periodontal ligament (Figure 2A,B) and found significantly more (*p* < 0.05) osteoclasts in naive dKO mice at both nine and 12 months compared with control and ibuprofen-treated dKO mice (Figure 2C).

### 2.3. Ibuprofen Alleviated Histomorphological Abnormalities in the Periodontal Tissue of dKO Mice

**Figure 3:**
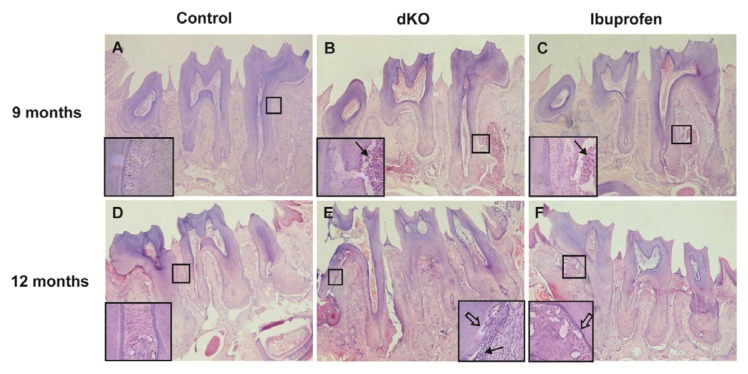
Histological observation of the periodontium (HE stained, 40× and 100×) Histological observations of control mice showed normal periodontal tissue structure (**A**,**D**); In naive dKO mice, periodontal tissues were infiltrated with inflammatory cells (black arrows) and the cementum became irregular (white arrow) (**B**,**E**); These abnormalities were partially absent after ibuprofen treatment (**C**,**F**).

In periodontitis, the inflammatory process extends from the gingiva to deeper connective tissues, resulting in the loss of connective tissue, bone, and finally teeth. Mild cementum and alveolar bone resorption causes an irregular increase in the width of the periodontal ligament due to an uneven reduction in the cortical plate in the bone and cementum layer [10]. In this study, periodontal ligament width in nine-month-old control mice was consistent and the cementum and alveolar bone was arranged normally. In contrast, periodontal ligament width in 12-month-old naive dKO mice was larger, the fibers of the periodontal ligament were fewer, and the cementum became irregular (Figure 3, white arrows). Inflammatory factors invaded the root of first molar (Figure 3, black arrows). After treatment with ibuprofen for six months, the abnormalities in periodontal tissue were partially absent (Figure 3F).

### 2.4. Inflammatory Mediators in the Brain and Gingiva Were Reduced with Ibuprofen Treatment

**Figure 4:**
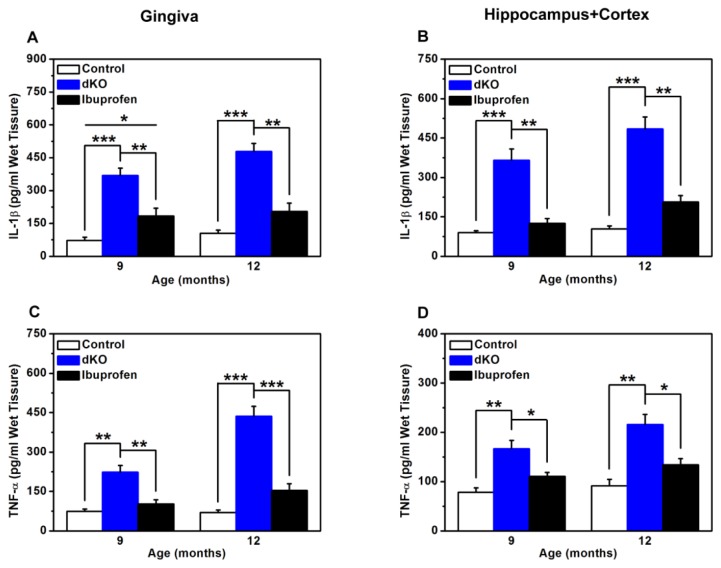
ELISA measurements of IL-1β (**A**,**B**) and TNF-α (**C**,**D**) from control, naive dKO, and ibuprofen-treated dKO mice at nine and 12 months. (**A**,**C**) Gingiva of the incisor teeth. (**B**,**D**) Hippocampus and cortex. *p < 0.05, **p < 0.01, ***p < 0.001.

IL-1β and TNF-α levels in the gingiva, hippocampus, and cortex of naive dKO mice were significantly higher compared with nine- and 12-month-old control mice (Figure 4A–D). These levels were dramatically lower in the ibuprofen-treated group (Figure 4A–D). Noteworthy, average IL-1β levels in gingiva were significantly higher in the ibuprofen-treated group compared with the control group at nine months (Figure 4A). The results were normalized to wet tissue weight.

### 2.5. COX-2 and PGE2 Levels Were Significantly Lower after Ibuprofen Treatment

**Figure 5:**
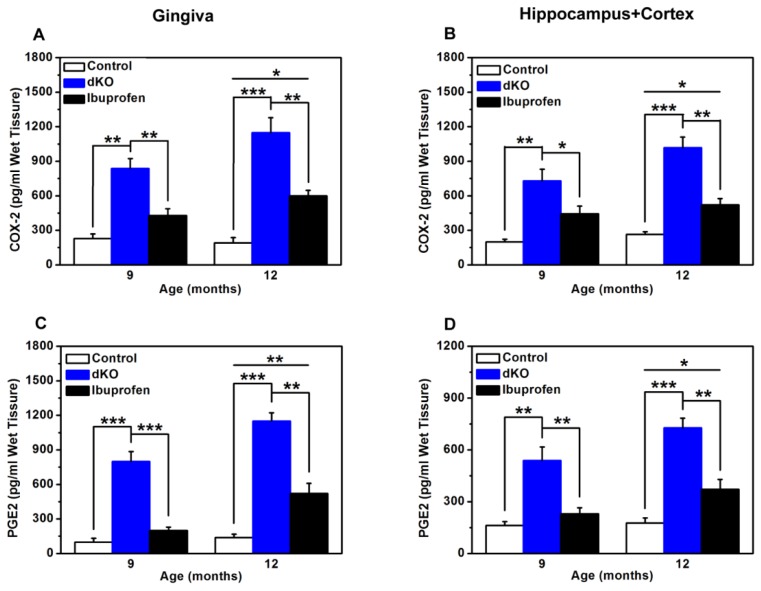
ELISA measurements of COX-2 (**A**,**B**) and PGE2 (**C**,**D**) in control, naive dKO, and ibuprofen-treated dKO mice at nine and 12 months. (**A**,**C**) Gingiva of the incisor teeth. (**B**,**D**) Hippocampus and cortex. *p < 0.05, **p < 0.01, ***p < 0.001.

At both nine and 12 months, COX-2 and PGE2 levels were significantly higher in naive dKO mice compared with control mice (Figure 5A–D). In the ibuprofen-treated group, both COX-2 and PGE2 levels were dramatically lower than in the naive dKO group (Figure 5A–D). However, average COX-2 and PGE2 levels were significantly higher in the ibuprofen-treated group than in the control group at 12 months (Figure 5A–D).

Our colleagues have found that the inflammatory response appeared in *PS* dKO mice at three months [7] and then spread to the periphery at six months, including the oral tissues, which can ultimately induce abnormalities in periodontal tissues [19]. Periodontal tissues (tooth-supporting tissues) include the gingiva, alveolar bone, cementum, and periodontal ligament. The periodontal ligament is located between the alveolar bone and the cementum [27]. The inflammatory process of periodontitis extends from the gingiva to deeper connective tissues, and severe resorption leads to tooth loss. Mild resorption of cementum and alveolar bone results in an irregular increase in the width of the periodontal ligament because the cortical plate of the alveolar bone and the cementum layer decrease unevenly [27]. In this study, histological observations of control mice showed normal periodontal tissue structure, while in naive dKO mice the periodontal ligament was irregular and thick, and the cortical plate and cementum layer were inconsistent and irregular (Figure 3). Alveolar bone height decreased with age in both control and naive dKO mice. However, when compared with control mice, the rate of alveolar bone loss in naive dKO mice was much faster and more severe (Figure 1). Furthermore, we found a significantly higher number of osteoclasts in the periodontal ligament of naive dKO mice than in control mice at nine and 12 months (Figure 2C). These results verified the periodontal tissue abnormalities in naive dKO mice found in our previous studies. As mentioned in the introduction, people with impaired cognition might be inattentive to oral hygiene or oral health maintenance, which results in poorer dental health [28]. Since no mice received oral care treatment in this study, our results indicate that inflammatory response is the most likely cause of the periodontal tissue abnormalities found in dKO mice.

In studies regarding the progression of periodontitis, ibuprofen has been one of the most frequently used anti-inflammatory drugs [23]. In this study, three-month-old and six-month-old dKO mice were fed chow that contained ibuprofen (375 ppm) for six months. We found that ibuprofen alleviated the abnormalities in periodontal tissues: the number of inflammatory cells in the periodontal ligament was less and the cementum layer was consistent and regular (Figure 3), and alveolar bone loss was significantly less at 12 months (Figure 1C). Moreover, significantly fewer osteoclasts were found at nine and 12 months (Figure 2C), which was caused by less alveolar bone loss and a thinner periodontal ligament. Note, however, that here fewer osteoclasts is not an indication of alveolar bone loss because we only counted osteoclasts in periodontal ligaments, the area of which decreased substantially due to severe periodontitis. Periodontal diseases are most often caused by the interaction between specific bacteria and components of the host immune response, while the inflammation found in dKO mice is the result of infection. These findings support the idea that periodontal abnormalities in naive dKO mice are due to the severe inflammatory response that originates from brain and subsequently spreads to the periphery, and that long-term ibuprofen treatment could markedly alleviate these abnormalities through its anti-inflammatory effects.

Our colleagues have conducted another study in which ibuprofen inhibited the activation of microglia which had caused release of inflammatory cytokines such as IL-1β and TNF-α in the brain of naive dKO mice [29]. Transgenic mice over-expressing TNF-α, or its receptors, in the brain develop chronic inflammation and neurodegeneration [30]. IL-1β has been reported to increase the expression of amyloid precursor protein (APP) in neuronal culture [31], and exposure of primary neurons to IL-1β exacerbates tau phosphorylation [32]. Therefore, we checked for IL-1β and TNF-α to assess the inflammatory response in naive dKO mice. Our results showed that levels of these inflammatory cytokines were higher not only in the brain but also in periodontal tissues in naive dKO mice at nine and 12 months (Figure 4), further confirming that periodontal tissue abnormalities resulted primarily from inflammatory responses in dKO mice.

Non-steroidal anti-inflammatory drugs (NSAIDs) have received considerable attention as a potential therapy both for preventing alveolar bone resorption in the progression of periodontitis [23] and for AD [22,33]. NSAIDs are typically used to reduce inflammatory response by inhibiting COX isoforms. COX-1 is constitutively expressed in most tissues and generates protective prostanoids that are pro-inflammatory and that maintain the gastric mucosa. Expression of COX-2 is commonly induced with growth factors, tumor promoters, hormones, bacterial endotoxin, cytokines, anoxia, neurotoxins, electrical stimulation, and pro-inflammatory stimuli [34,35]. Therefore, it has been accepted that COX-1 is constitutively expressed whereas COX-2 is expressed as part of the inflammatory response in most tissues. Being the prostanoid most generally associated with inflammatory responses, the formation of prostaglandin E2 (PGE2) at inflammation sites is often taken as an indicator of local COX activity and its inhibition as an index of the ability of NSAIDs or newer COX-2-selective agents to inhibit COX-2. Ibuprofen, as one of the most frequently used NSAIDs, may reduce the severity of inflammatory responses in both periodontitis and AD [23,36]. In our study, dKO mice treated with ibuprofen showed significantly lower levels of COX-2 and its metabolite PGE2 in the brain and periodontal tissue compared with non-treated dKO mice (Figure 5). These data confirmed that ibuprofen likely has a role in ameliorating both CNS inflammation and peripheral inflammation.

Based on their data, Akiyama and Hoozemans have suggested that NSAID-mediated COX inhibition might reduce inflammation *in vivo* by interrupting a putative feed-forward inflammatory mechanism [37,38]. Primary cell-culture experiments have shown that pro-inflammatory cytokines, such as IL-1β and TNF-α, can up-regulate COX-2 expression and PGE2 in neurons, astrocytes, and microglia. Conversely, enhanced expression of COX-2 and PGE2 can increase the production of cytokines, prostanoids, and mediators of oxidative stress [37,38]. Our data showed a significant increase of COX-2 and PGE2 in naive dKO mice. Results proved that the pro-inflammatory cytokines IL-1β and TNF-α induced increases in the pro-inflammatory molecule COX-2 and its metabolite PGE2. We therefore examined the level of IL-1β and TNF-α in the brain and periodontal tissue. Compared with those in naive dKO mice, the levels of IL-1β and TNF-α were significantly lower in the brain and periodontal tissue of ibuprofen-treated mice. These results proved that reduction of inflammation caused by NSAID-mediated COX inhibition is through the putative feed-forward inflammatory mechanism.

Our data show that ibuprofen attenuated inflammation in the brain and in periodontal tissue in naive dKO mice at nine and 12 months. As mentioned above, a relatively straightforward argument was made for cognitive impairment leading to poor dental health: People with impaired cognition might be inattentive to oral hygiene or oral health maintenance as impairment in cognition progresses [28]. Our colleagues have found that ibuprofen partly alleviates learning and memory deficits in dKO mice [29]. We deduce that ibuprofen treatment might improve impaired cognition, including maintenance of good oral habits that is beneficial to oral hygiene and prevention of periodontitis. Moreover, reduction of brain inflammation via ibuprofen might help to decrease peripheral inflammation, such as periodontitis. In conclusion, ibuprofen could attenuate brain and periodontal inflammation, and these improvements might interact with each other to form a virtuous cycle.

## 3. Experimental Section

### 3.1. Mice Breeding and Genotyping

Breeding and genotyping of *presenilin*s (*PS*) dKO mice have been described previously [5,6,39]. Briefly, conditional *PS* dKO mice were obtained by crossing forebrain-specific *PS1* heterozygous knockout mice with conventional *PS2* heterozygous knockout mice on a B6/CBA F1 genetic background. Mice with the Cre transgene, f*PS1*/f*PS1* and *PS2*^−/−^ served as dKO, their littermates (no Cre, f*PS1*^−/+^ and *PS2*^+/+^ or f*PS1*^−/+^ and *PS2*^+/−^) served as controls. dKO mice were fed either standard chow (naive dKO) or standard chow containing 375 ppm ibuprofen (ibuprofen-treated), the same dosage that has been used in previous studies of AD-model mice [24]. Treatment of the mice started at three and six months of age and lasted for six months, generating the nine and 12-month-old groups, respectively. All mice were housed at 20–26 °C and in 40%–70% humidity with 12 h light/dark cycles, and were sacrificed at the age of nine and 12 months. All experiments were approved by the Animal Ethics Committee of East China Normal University, China.

### 3.2. Immunohistochemical Analysis of the Calcitonin Receptor in the Periodontal Ligament

Immunohistochemical analysis was conducted following methods described in Han *et al.* [19]. Sections (5 μm) of the upper molars were mounted onto amino-propyl-tri-ethoxy-silane (APES)-coated slides (Shanghai Biochip, Shanghai, China). After dewaxing in xylene, sections were dehydrated in ethanol and rinsed in distilled water for 10 min. Antigen retrieval was performed by immersing slides in 0.1% trypsin (BIOS, Beijing, China) and 0.01 M Tris-buffered saline (TBS, pH 7.2) for 30 min, and endogenous peroxidase activity was blocked with 3% *v*/*v* H_2_O_2_ in water for 3 min. For calcitonin receptor (CTR) detection, sections were incubated with a rabbit polyclonal anti-CTR primary antibody (bs-0124R, BIOS, Beijing, China) at 1:100 dilutions for 60 min at 37 °C. For negative controls, the primary antibody was replaced with rabbit serum. The standard streptavidin-biotin-peroxidase complex method was performed to detect the primary antibody with the use of a Histostain-Plus Kit (BIOS, Beijing, China). Sections were rinsed in phosphate-buffered saline for 10 min, processed with a Diaminobenzidine Substrate-Chromogen Kit (BIOS, Beijing, China), counterstained in Meyer’s hematoxylin, and covered with a neutral balsam-mounting medium. Sections were examined by light microscopy (Olympus, Osaka, Japan). Five sections, at least 25 μm apart, were evaluated from each animal (7–9 animals in each group). Following thorough examination of the section (400× magnification), staining was considered to be intense when 50% or more of the cells were stained. The stained cells in the periodontal ligament were counted with a computer-assisted image-analysis system (Image-pro Plus 6.0, Media Cybernetics, Silver Spring, MD, USA, 2006). The total number of stained cells found in five sections from each animal was used for statistical analysis.

### 3.3. Histological Observation of Periodontal Tissues

Histological observation was conducted following methods described in Han *et al.* [19] Maxillae with three upper molars were immersed in fixative composed of 4% paraformaldehyde in phosphate-buffered saline for 4 h at 4 °C. After the fixation, the specimens were demineralized in 10% EDTA for 3 weeks. The solution was changed every 2–3 days. The demineralized specimens were then dehydrated in a graded series of ethanol and embedded in 60–62 °C paraffin. The paraffin sections measuring 5 μm in thickness were cut and stained with hematoxylin and eosin. All slides were comparatively evaluated using an Olympus BH2 light microscope (40× and 100×) (Olympus, Osaka, Japan).

### 3.4. Morphometric Bone Observation

Morphometric bone observation was conducted following methods described in Han *et al.* [19]. The mandibles with three lower molars were defleshed and stained with 1% methylene blue to delineate the cementoenamel junction. The buccal and lingual surfaces were digitally imaged using a calibrated dissecting microscope (30×) (Nikon, New York, NY, USA). The captured images were then further analyzed using a computer-assisted image analysis system (Image-pro Plus 6.0, Media Cybernetics, Silver Spring, MD, USA, 2006) by an independent investigator who was unaware of the treatment regimes. The height of alveolar bone loss was calculated as the sum of the exposed molar root area (from the cementoenamel junction to the alveolar bone crest) for both buccal and lingual aspects from the left and right sides.

### 3.5. Enzyme-Linked Immunosorbent Assay (ELISA) for Inflammatory Mediators

ELISA was conducted following methods described in Han *et al.* [19]. ELISA kits for interleukin-1β (IL-1β) and tumor necrosis factor-α (TNF-α) were purchased from R & D Systems, USA. Two additional ELISA kits for cyclooxygenase (COX-2) and prostaglandin E2 (PGE2) were purchased from Invitrogen (Minneapolis, MN, USA). All assays were performed according to the manufacturer’s instructions. Briefly, 96-well plates were separately coated with the antibodies and blocked. Gingiva of mandibular incisor teeth were homogenized on ice in RIPA Lysis Buffer (Beyotime, Jiangsu, China) followed by centrifugation at 12,000× *g* at 4 °C. Supernatants were loaded into the wells and incubated at 37 °C for 60 min. After washing, the plates were incubated with biotinylated antibodies. Plates were then washed four times followed by alkaline phosphatase treatment. After washing the plate, substrate was added to each well for 15 min to allow color development. The reaction was stopped by adding 50 μL 0.5 M H_2_SO_4_ to each well. Absorbance was read at 450 nm (Tecan GENios, Port Melbourne, Victoria, Austria). Concentrations were normalized to the loaded amount of wet tissue. All experiments were conducted in triplicate.

### 3.6. Statistical Analysis

One-factor ANOVAs were performed to analyze differences in the number of osteoclasts in the periodontal ligament, the area of root exposure, and the inflammatory mediators COX-2 and PGE2 in periodontal tissue, the hippocampus, and cortex. *p*-values < 0.05 were considered significant.

## 4. Conclusions

Our study provided further evidence for the impact of AD on periodontal tissues. The forebrain-specific *PS1*/*PS2* conditional double knockout in mice led to abnormalities in periodontal tissues at the age of nine and 12 months. Ibuprofen, as a commonly used nonprescription NSAID associated with reduced AD and periodontitis risk in epidemiological studies, not only improved the abnormalities in periodontal tissues but also attenuated inflammatory responses in the brain and periodontal tissue in dKO mice at both nine and 12 months. These effects were most likely mediated by the inflammatory cyclooxygenase pathway. The improvement in periodontal tissue abnormalities resulting from ibuprofen treatment could be helpful for oral health maintenance, as well as slow down the progression of periodontal disease due to brain inflammation.
